# N-Terminal Pro B-Type Natriuretic Peptide's Usefulness for Paroxysmal Atrial Fibrillation Detection Among Populations Carrying Cardiovascular Risk Factors

**DOI:** 10.3389/fneur.2019.01226

**Published:** 2019-11-29

**Authors:** Elena Palà, Alejandro Bustamante, Josep Lluis Clúa-Espuny, Juan Acosta, Felipe Gonzalez-Loyola, Juan Ballesta-Ors, Natalia Gill, Andrea Caballero, Jorge Pagola, Alonso Pedrote, Miguel Angel Muñoz, Joan Montaner

**Affiliations:** ^1^Neurovascular Research Laboratory, Vall d'Hebron Institute of Research (VHIR)-Universitat Autónoma de Barcelona, Barcelona, Spain; ^2^Department of Neurology, Hospital Universitari Vall d'Hebron, Barcelona, Spain; ^3^Equip d'Atenció Primària Tortosa Est, SAP Terres de l'Ebre, Institut Català de la Salut, Tortosa, Spain; ^4^Unitat de Suport a la Recerca de Barcelona, Institut d'Investigació en Atenció Primària IDIAP Jordi Gol, Barcelona, Spain; ^5^Department of Cardiology, Hospital Universitario Virgen del Rocio, Sevilla, Spain; ^6^Gerència Atenció Primària de Barcelona, Institut Català de la Salut, Barcelona, Spain; ^7^Biochemical Department, Hospital Universitari Vall d'Hebron, Barcelona, Spain

**Keywords:** stroke, atrial fibrillation, biomarker, screening, NT-proBNP

## Abstract

**Background:** Atrial fibrillation (AF) systematic screening studies have not shown a clear usefulness in stroke prevention, as AF might present as paroxysmal and asymptomatic. This study aims to determine the usefulness of some blood-biomarkers to identify paroxysmal atrial fibrillation in the context of a screening programme.

**Methods:** A total of 100 subjects aged 65–75 years with hypertension and diabetes were randomly selected. AF was assessed by conventional electrocardiogram (ECG) and 4 weeks monitoring with a wearable Holter device (Nuubo™). N-terminal pro B-type natriuretic peptide (NT-proBNP), apolipoprotein CIII (ApoC-III), von Willebrand factor (vWF), ADAMTS13, urokinase plasminogen activator surface receptor (uPAR), and urokinase plasminogen activator (uPA) were determined in serum/plasma samples and the levels were compared depending on AF presence and mode of detection.

**Results:** The AF prevalence in the studied population was found to be 20%. In seven subjects, AF was only detected after 1 month of Holter monitoring (hAF group). NT-proBNP levels were higher in subjects with AF compared with subjects with no AF (*p* < 0.0001), even when only taking into account the hAF group (*p* = 0.031). No significant differences were found in the other biomarkers. The NT-proBNP >95 pg/ml cut-off showed high sensitivity and specificity to detect AF (95%, 66.2%) or hAF (85.72%, 66.2%) and was found to be an independent predictor of AF and hAF in a logistic regression analysis. NT-proBNP correlated with AF burden (*r* = 0.597, *p* = 0.024).

**Conclusion:** NT-proBNP was elevated in AF cases not identified by ECG; thus, it may be used as a screening biomarker in asymptomatic high-risk populations, with a promising cut-off point of 95 pg/ml that requires further validation.

## Introduction

Atrial fibrillation (AF) is one of the fastest-growing cardiovascular epidemics of the twenty-first century (1). This heart rhythm disorder multiplies by five the risk of ischemic stroke (2), and its prevalence in the population over 60 years has been quantified at 10.9% (3). Anticoagulation may reduce stroke risk in AF individuals by ~60% (4), but unfortunately, given its paroxysmal and asymptomatic nature at earlier stages, AF is often undiagnosed and/or undertreated when stroke occurs. In a previous study, the absolute prevalence of undiagnosed AF in individuals over 60 years was 3.4%, representing up to 31% of the overall AF prevalence in our area (5, 6). International collaborations (7, 8) recommend opportunistic screening for AF in patients >65 years as a strategy to reduce stroke and death, but systematic screening programmes have failed to show a clear benefit and those strategies are lacking in high-risk populations (9–11).

Blood biomarkers might represent an alternative tool for AF screening, because some of them might have elevated circulating levels after AF paroxysms, a kind of “biological memory” offering a temporal advantage over electrical detection of the paroxysm itself. N-terminal pro B-type natriuretic peptide (NT-proBNP) has been found to be elevated in cardioembolic (CE) stroke (12) and to be associated with AF (13–16). Von Willebrand factor (vWF) was found to be elevated in non-valvular AF (17) and, together with apolipoprotein CIII (ApoC-III), was found to be predictor of stroke CE etiology [unpublished results from the Stroke-Chip study (18)]. ADAMTS-13 is the key regulator of vWF, and it has been associated with AF recurrence after cardioversion (19). Urokinase plasminogen activator surface receptor (uPAR) has been associated with AF prevalence (20) while its ligand, urokinase plasminogen activator (uPA), has not been studied in AF patients.

This study aims to determine the usefulness of the aforementioned biomarkers to identify AF individuals in the context of a screening programme, especially in cases of paroxysmal AF that would not be detected using a regular 12-lead ECG.

## Materials and Methods

### Patients and Protocol

AFRICAT (Atrial Fibrillation Research in CATalonia; NCT03188484) is an observational, multicenter, population-based study with the aim of developing a screening programme to detect new cases of AF in high-risk individuals in primary care centers. In Phase I of the project, 100 subjects aged 65–75 with hypertension and diabetes were selected from primary care registers (e-cap), which include the clinical records from most Catalonia inhabitants between January 2016 and December 2017. In two different health service areas, Servei d'Atenció Primària (SAP) Muntanya and SAP Terres de l'Ebre, patients fulfilling the inclusion criteria were identified in primary care centers from each catchment [Centre d'Atenció Primària (CAP) Sant Rafael and CAP Trinitat Vella in SAP Muntanya; Equip d'Atenció Primària (EAP) Tortosa Est and EAP Tortosa Oest in SAP Terres de l'Ebre]. Physicians from these primary care centers were informed about the study protocol, and patients were invited by them to participate in the study until 50 patients were recruited in each catchment. Participants were ambulatory patients in a basal situation, meaning they were contacted at home to arrange a visit in their primary care center specifically for the study. During the appointments, patients received a comprehensive assessment consisting of clinical characteristics (demographic factors, vascular risk factors, medications, comorbidities, and vitals), electrographic assessment and a blood sample collection. Comorbid conditions were defined using standard outpatient and inpatient ICD-10 codes by electronic data capture, including pharmacy records, laboratory data, and emergency room and hospitalization diagnoses across all primary care centers and hospitals.

Individuals with chronic inflammatory diseases, cancer or dementia were excluded. AF was assessed by conventional electrocardiogram (ECG). Moreover, patients were monitored for 4 weeks with a wearable Holter device (Nuubo^TM^) as described previously (21). The Holter device was composed of a sensor that captured the electrocardiographic signal by non-invasive textile electrodes and a recorder of the signal that stored the information for subsequent detailed analysis. Participants were instructed by local trained researchers to wear the Holter for 23 h and recharge it for 1 h daily. After 4 weeks, individuals brought the Holter to their primary care center, and data were collected. Holter records, anonymized and encrypted, were sent together with the ECG for blinded reading to the Rhythm Disorders Unit at the Cardiology Department of Hospital Virgen del Rocio in Seville, to verify AF episodes. AF was defined following AHA guidelines as irregular R-R intervals without a P wave signal, lasting for more than 60 s (22). Individuals with previous AF were not excluded at this step, but Holter monitoring was optional in this subgroup.

Patients were classified and compared in five groups defined by past medical history (PMH) for AF, ECG findings and Holter AF detection as follows: no AF, PMH-ECG-Holter+ [Holter-detected AF (hAF)], PMH-ECG+, PMH+ECG-, and PMH+ECG+. Specific comparisons were done between the hAF and no AF groups.

The AFRICAT study protocol was approved by the clinical research ethics committees of IDIAP Jordi Gol (P15/047) and Hospital Universitari Vall d'Hebron [PR (AG) 133-2015]. All participants signed informed consent before inclusion. The study protocol conformed to the ethical guidelines of the 1975 Declaration of Helsinki.

### Biomarker Measurement

Blood was collected into EDTA and serum tubes at the time of inclusion. After centrifugation at 1,500 g and 4°C for 15 min, plasma and serum aliquots were frozen at −80°C until biomarker determination.

Plasma NT-proBNP levels were determined by automated immunoassay in a COBAS c8000 (Roche Diagnostics); serum ApoC-III, plasma uPAR and plasma uPA by ELISA (Abnova, R&D Systems, and Cloud-Clone); and plasma vWF and ADAMTS13 by a magnetic Luminex® assay (R&D Systems).

All assays were performed blinded to clinical information and according to the manufacturer's instructions. All samples were tested in duplicate and inter-assay variation was determined by a commercial control (Human Serum, male AB, USA origin from clotted, SIGMA, ref number H16914; Human plasma K2 EDTA, Innovative Research, ref number IPLA-N) tested in duplicate in each plate. When inter-assay variation was >20% biomarker levels were standardized using the Z-score.

### Statistical Analysis

Statistical analysis was conducted with SPSS version 20. Graphs were elaborated with GraphPad Prism 6. Data are expressed as number (%) for categorical variables and as mean ± SD or median (interquartile range) for continuous variables, depending on the data distribution. For univariate analysis, the Mann–Whitney *U*-test or Student's *t*-test was used for continuous variables, and the χ^2^ test was used for categorical variables. ANOVA or the Kruskal–Wallis test was used to compare >2 variables depending on variable distribution. Receiver operator characteristic (ROC) curves were configured to calculate sensitivity, specificity, and cut-off values. The Spearman test was used for correlations. Comparisons were first performed between AF patients and no AF patients and second between hAF and no AF patients (excluding other subtypes of AF). Binary logistic regression analysis was performed including variables associated with AF or hAF in univariate analysis.

## Results

### Patient Characteristics and AF Detection

Clinical characteristics of the cohort can be found in Table 1. Coronary heart disease (*p* = 0.008), heart failure (*p* = 0.007), valvular diseases (*p* = 0.024), and use of anticoagulants (*p* < 0.0001) were more common in AF individuals. Of the 9 anticoagulated individuals, only one was anticoagulated for reasons other than AF (ischemic cardiopathy and deep venous thrombosis). The CHA2DS2-VASc score was higher in AF individuals (*p* = 0.035). Coronary heart disease ischemia was more common in hAF individuals compared to no AF (*p* = 0.032). No cases of heart failure, valvular disease or anticoagulation were present in this subgroup.

**Table 1:** Clinical characteristics of the cohort and comparison according to atrial fibrillation diagnosis.

	**All patients (100)**	**AF (20)**	**No AF (80)**	**hAF (7)**	***P*-value^a^**	***P*-value^b^**
Age	70 (68–73)	69 (66–71.5)	70 (68–73)	70 (65–72)	0.273	0.655
Sex (% female)	33 (33)	7 (35)	26 (32.5)	3 (42.9)	0.832	0.682
Tobacco	20 (20)	4 (20)	16 (20.3)	1 (14.3)	1.000	1.000
Alcohol	11 (11)	1 (5)	10 (12.5)	0 (0)	0.456	1.000
Dyslipidaemia	81 (81)	16 (80)	65 (81.3)	5 (71.4)	1.000	0.619
Coronary heart disease	18 (18)	8 (40)	10 (12.5)	3 (42.9)	0.008^*^	0.032^*^
Heart failure	3 (3)	3 (15)	0 (0)	0 (0)	0.007^*^	1.000
Valvular disease	4 (4)	3 (15)	1 (1.3)	0 (0)	0.024^*^	0.767
Previous stroke	6 (6)	2 (10)	4 (5)	0 (0)	0.597	1.000
Anticoagulation	9 (9)	8 (40)	1 (1.3)	0 (0)	0.000^*^	0.920
Antiplatelets	50 (50)	7 (35)	43 (53.8)	3 (42.9)	0.134	0.702
Familiar history FA	5 (5)	2 (10)	3 (3.9)	0 (0)	0.273	1.000
SBP, mm Hg	143.50 (134–153)	140.5 (127.5–162.5)	144 (134–151.75)	140 (124–168)	0.973	0.773
DBP, mm Hg	78.95 ± 10.09	80.95 ± 10.85	78.45 ± 9.90	79.14 ± 6.66	0.332	0.857
Heart rate	77.94 ± 15.70	77.25 ± 16.94	78.11 ± 15.48	73 ± 22.14	0.724	0.421
CHA_2_DS_2_-VASc	4 (3–4)	4 (3–4)	4 (3–5)	4 (3–5)	0.035^*^	0.284

a *P-value comparison AF vs. no AF*.

b *P-value comparison hAF vs no AF*.

* *P < 0.05. AF, atrial fibrillation; DBP, diastolic blood pressure; hAF, Holter-detected atrial fibrillation; SBP, systolic blood pressure*.

Of the included 100 individuals, 96 were monitored with ECG Holter. All patients with negative ECG were monitored. Median monitoring time was 457 h. Six cutaneous adverse events related to wearing the Holter were noted; these consisted of a rash that normally disappeared with topical steroids. However, in two subjects, these adverse events prevented the monitoring being continued.

**Figure 1:**
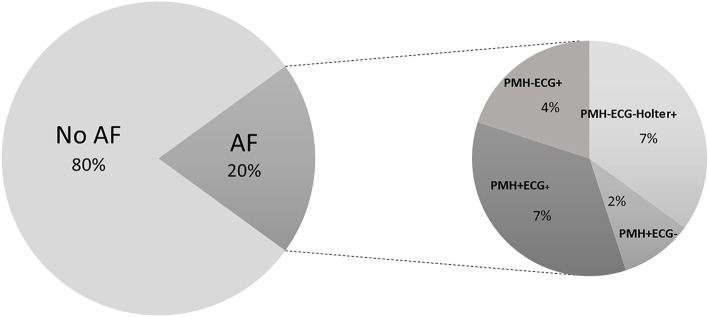
Subject classification. Individuals were classified depending on AF presence and subgroups were made depending on AF detection with different methods and previous medical history.

Of the 100 selected subjects, AF was present in 20 (11 newly detected within the present study) classified as follows: 7 PMH-ECG-Holter+ (hAF), 4 PMH-ECG+, 2 PMH+ECG-, and 7 PMH+ECG+ (Figure 1).

In 14 individuals (11 newly detected and 3 PMH+ECG+ were monitored), AF was detected during Holter monitoring and AF burden was calculated. AF burden was defined as minutes being in AF divided by the total minutes of readable records and is expressed as a percentage. The median AF burden was 10.35% (1.00–56.50).

### Biomarker Analysis

NT-proBNP, ApoC-III, sUPAR, vWF, and ADAMTS13 inter- and intra-assay variation was acceptable (coefficient of variation <20); hence, all the samples were included in the analysis. uPA levels were standardized because the inter-assay variation was higher than 20%, and 9 samples were eliminated from statistical analysis because their duplicates showed CV > 20%. Median levels and comparisons are shown in Table 2.

**Table 2:** Biomarker levels and comparisons between different groups.

	**AF (20)**	**No AF (80)**	**hAF (7)**	***P*-value^a^**	***P*-value^b^**
NT-proBNP (pg/ml)	643.65 (155.72–1339.25)	64.28 (37.08–133.10)	128.30 (97.69–191.8)	<0.0001^*^	0.03^*^
ApoC-III (ng/ml)	107,594 (92,501–129262.06)	104,450 (766,64–130,393)	105,352 (91,956–132,268)	0.496	0.574
ADAMTS13 (ng/ml)	1131.24 ± 471.419	1221.41 ± 459.80	1271.68 ± 561.01	0.437	0.786
vWF (ng/ml)	0.139 ± 0.052	0.139 ± 0.706	0.146 ± 0.060	0.987	0.802
uPA (std)^c^	2.01 (1.41–2.39)	1.60 (1.38–2.35)	1.68 (1.29–2.35)	0.720	0.868
uPAR (pg/ml)	2220.80 (557.74–2810.52)	2267.54 (1518.33–2763.05)	2246.12 (1926.03–259.23)	0.689	0.918

a *P-value comparison AF vs. no AF*.

b *P-value comparison hAF vs. no AF*.

c *Ninety-one individuals included in the uPA analysis (73 no AF and 18 AF, of whom 6 were hAF)*.

* *P < 0.05. AF, atrial fibrillation; ApoC-III, Apolipoprotein C-III; hAF, Holter-detected group; NT-proBNP, N-terminal pro B-type natriuretic peptide; vWF, von Willebrand factor; uPA, urokinase plasminogen activator; uPAR, urokinase plasminogen activator surface receptor*.

#### AF vs. No AF

**Figure 2:**
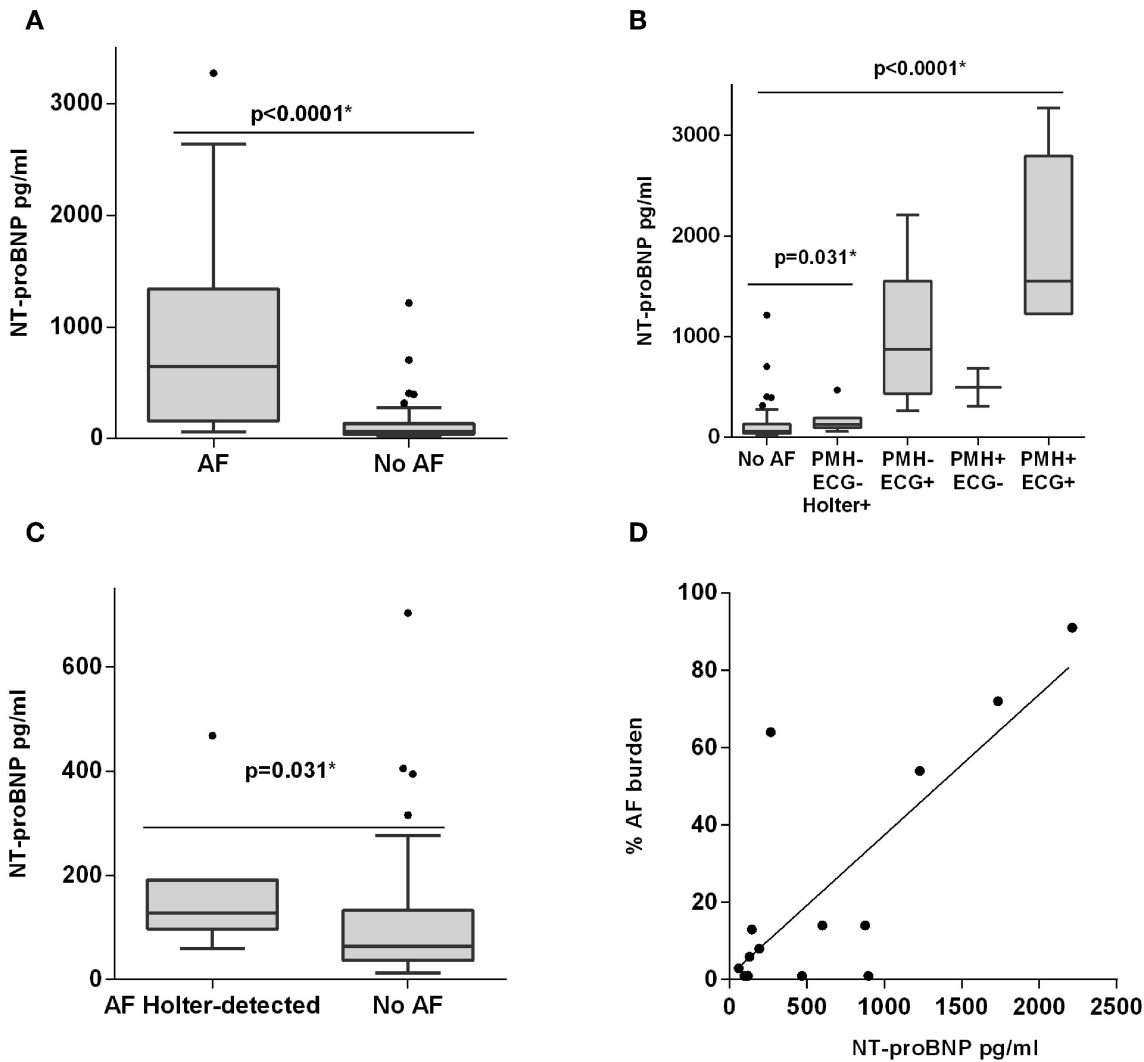
Plasma NT-proBNP level distribution. **(A)** Boxplot distribution between AF and no AF. **(B)** Boxplot distribution between different groups depending on past medical history (PMH) for AF, ECG findings, and Holter monitoring. **(C)** Boxplot distribution between Holter-detected AF (hAF) and no AF. **(D)** Correlation between NT-proBNP levels and AF burden. *P < 0.05.

**Figure 3:**
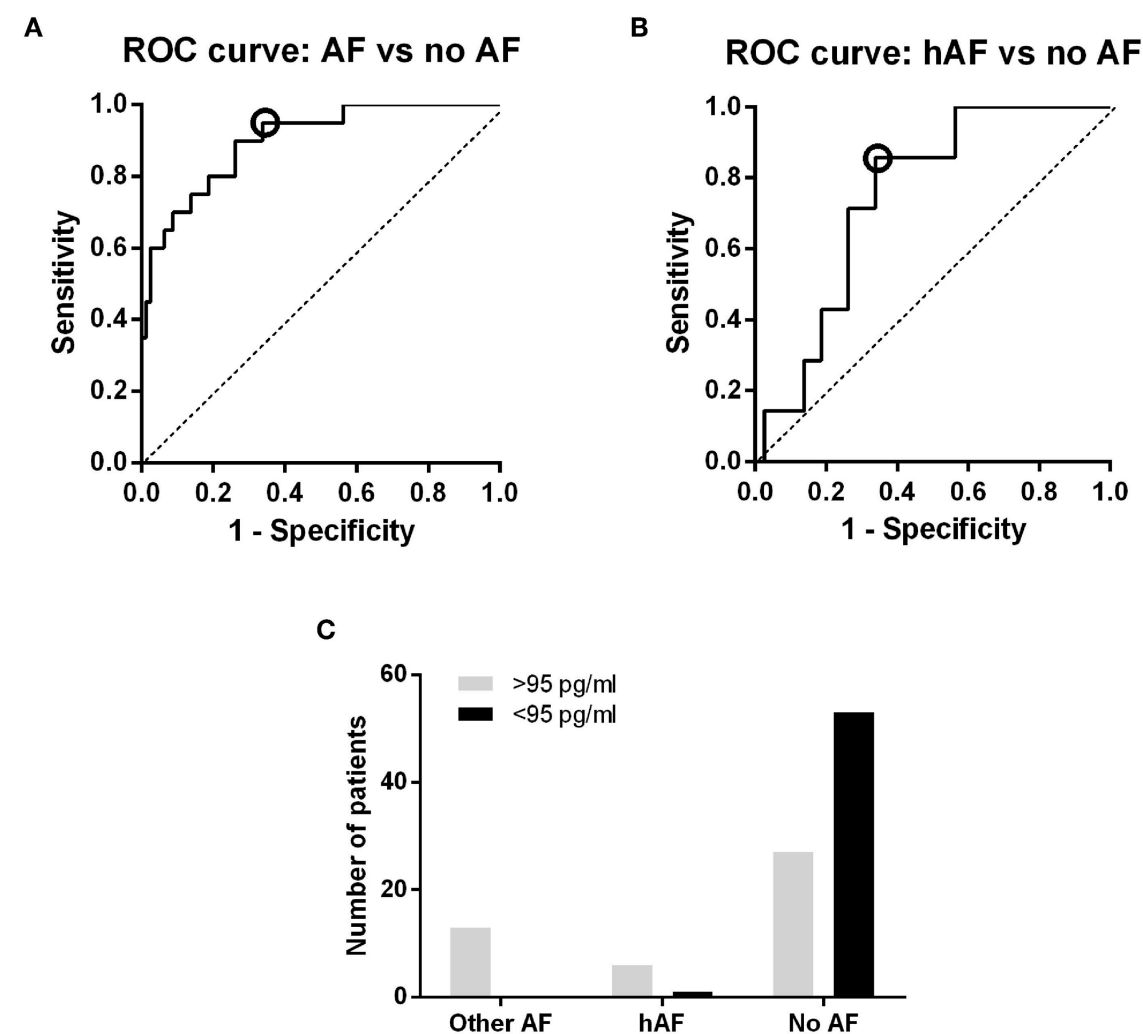
NT-proBNP discrimination power. **(A)** ROC curve: ability to discriminate between AF and no AF. **(B)** ROC curve, ability to discriminate between Holter-detected AF and no AF. The cut-off with the best specificity and sensitivity to detect hAF is marked with a circle in the two curves (95 pg/ml), as it is the cut-off value that was used to calculate sensitivity, specificity and predictive values. **(C)** Discrimination power of the NT-proBNP cut-off value of 95 pg/ml. All AF individuals had NT-proBNP plasma levels below the previous cut-off (except for a patient with paroxysmal AF), and the majority of no AF patients had values above the cut-off.

NT-proBNP was significantly higher in individuals with AF compared with no AF [643.65 pg/ml (IQR 155.72–1339.25) vs. 64.28 pg/ml (IQR 37.08–133.10), *p* < 0.0001; Figure 2A], while the remaining biomarkers were not different between the two groups (Supplementary Figures 1, 2). The NT-proBNP distribution across the different subgroups is shown in Figure 2B. In addition, AF burden was correlated with NT-proBNP (*r* = 0.597, *p* = 0.024; Figure 2D). The discriminating ability (area under the ROC curve) of NT-proBNP was 0.900 (95% CI, 0.8274–0.9726, *p* < 0.0001) to detect any AF (Figure 3A). The cut-off point of NT-proBNP of >95 pg/ml showed 95% sensitivity, 66.2% specificity, 41.3% positive predictive value (PPV), and 98.1% negative predictive value (NPV) to detect any AF (Figure 3C).

Anticoagulants (OR = 18.90; 95% CI, 2.10–169.83; *p* = 0.09) and NT-proBNP >95 pg/ml (OR = 22.42; 95% CI, 2.74–183.15; *p* = 0.04) were the only independent predictors of AF in the logistic regression analysis performed in the whole cohort. CHA2DS2-VASc was an independent AF predictor when considered alone in the regression analysis (OR = 2.29; 95% CI 1.16–4.44; *p* = 0.017). However, after the addition of NT-proBNP>95 pg/ml, CHA2DS2-VASc was not significant in the model.

Alternatively, the cut-off point of NT-proBNP of >125 pg/ml, which is recommended for heart failure diagnosis in the non-acute setting, showed 85% sensitivity and 73.8% specificity, 44.7% PPV and 95.2% NPV. NT-proBNP>125 pg/ml was also an independent predictor of AF (OR = 8.85; 95% CI, 2.17–35.95; *p* = 0.017).

Sensitivity analysis was performed while excluding the patient who was anticoagulated for reasons other than AF, and the results were similar.

#### hAF vs. No AF

NT-proBNP levels were different between no AF and hAF [64.28 pg/ml (IQR 37.08–133.10) vs. 128.3 pg/ml (IQR 97.69–191.3), *p* = 0.031; Figure 2C] while other biomarkers did not show differences (Supplementary Figures 1, 2). Patients without AF and with a Holter monitoring time below the median (457 h) were removed and all aforementioned comparisons were tested again, with similar results (data not shown). The discriminating ability (area under the ROC curve) of NT-proBNP was 0.7464 (95% CI 0.6087–0.8841, *p* = 0.031) to detect hAF (Figure 3B). The cut-off point of NT-proBNP of >95 pg/ml showed 85.7 % sensitivity, 66.2% specificity, 18.2% PPV, and 98.1% NPV to detect hAF (Figure 3C).

The logistic regression analysis performed on this subgroup confirmed that NT-proBNP ≥95 pg/ml was an independent predictor of Holter-detected atrial fibrillation (OR, 11.778; 95% CI, 1.35–102.85; *p* = 0.026). No clinical variable was an independent predictor of AF detection.

Alternatively, the cut-off point of NT-proBNP of >125 pg/ml showed 55.1% sensitivity, 73.8% specificity, 16% PPV, and 95.2% NPV. NT-proBNP >125 pg/ml also was an independent predictor of AF (OR = 8.85; 95% CI 2.17–35.95; *p* = 0.017). NT-proBNP >125 pg/ml was not an independent predictor of hAF.

## Discussion

The prevalence of AF in our population was 20%, of which 55% were newly detected AF individuals. This prevalence is higher than reported in previous screening studies (from 2.3 to 12.3%) (10, 11). Most such studies have only used ECG or pulse devices instead of long-term monitoring, so they have missed cases of paroxysmal AF. Moreover, we used a targeted screening approach, focussing on a high-risk population with advanced age and other conditions related to AF, such as hypertension and diabetes, who should present a higher AF prevalence (23). Longer monitoring in a high-risk population might therefore be a good strategy to further explore in larger cohorts. Other studies using long-term monitoring with insertable cardiac devices or pacemakers in patients without prior history of AF have also shown a higher prevalence of AF (24). In fact, in the recent REVEAL AF trial, detection rates of AF rose with the monitoring time (25). Moreover, in patients with cryptogenic stroke, long-term monitoring was found to be associated with higher rates of AF detection, with a significant impact in secondary prevention (26). However, although it could become the gold standard for AF detection, long-term monitoring devices are expensive and unsuitable for application in primary care. In that sense, biomarkers might be used as cost-effective alternatives for AF systematic screening.

The present study suggests that NT-proBNP might be used as a screening biomarker to detect even paroxysmal AF in asymptomatic, high-risk individuals. The focus of the present study was to assess the predictive ability of NT-proBNP and other biomarkers to diagnose paroxysmal atrial fibrillation and be used as the first step of a screening workflow. In fact, the best screening biomarker in this context would be able to detect those cases impossible to notice with other available tools, such opportunistic ECG or hand-held ECG devices. It should be mentioned that paroxysmal AF (pAF) patients also can have increased risk of stroke (27, 28). Moreover, pAF may progress to permanent AF within a year in almost 20% of cases in the aging population (29, 30).

Previous studies have suggested NT-proBNP as a screening tool (14–16), but a limited length of monitoring might have misclassified some cases of paroxysmal atrial fibrillation. Our study overcame this limitation by recording patient's heart rhythm continuously for 30 days using a Holter monitor, allowing us to detect more AF cases. This is to our knowledge the longest period over which asymptomatic individuals have been monitored to detect atrial fibrillation in a biomarker study, if we exclude studies using pacemakers (13). The limitation of pacemakers is that their use is restricted to very specific populations with cardiac diseases that could affect some biomarker levels.

In the pilot study of Seegers et al. (14), in addition to a limited 7-day Holter ECG for the evaluation of paroxysmal AF, patients were preselected as those with lowest and highest NT-proBNP quartiles, overestimating the discriminatory value of the biomarker. In our study, inclusion was performed before any biomarker determination, and 4-week Holter monitoring was used as the gold standard to assess the usefulness of blood-based biomarkers in AF screening. Although individuals with AF detected by other methods were not excluded from the whole study, to increase the statistical power, specific comparisons were performed while excluding these patients and only taking into account AF cases detected with long-term monitoring, i.e., cases with neither previous AF history nor detection during electrocardiographic assessment. Even though few patients were part of this group, specific comparisons showed the usefulness of NT-proBNP to identify those patients outside AF paroxysms. After validating those results with a larger cohort, NT-proBNP could be implemented as part of a screening programme to indicate long-term monitoring in patients with a risk biological profile. Such a protocol would identify patients at risk of AF who would benefit from anticoagulation, thereby improving primary stroke prevention by preventing cardioembolic stroke, which is associated with the poorest disability and mortality rates among stroke etiologies (31).

Additionally, we proposed for the first time a specific cut-off point for atrial fibrillation screening. Seegers et al. (14) suggested that NT-proBNP could be used for stroke prevention after finding increased values in AF individuals, without indicating any cut-off. Svenberg et al. (15) and Ghazal et al. (16) proposed a cut-off point of NT-proBNP >125 pg/ml, which is the recommended for heart failure in the non-acute setting (32). Here, NT-proBNP >95 pg/ml reached high sensitivity (95%) even to detect paroxysmal AF individuals (85.7%). In fact, in our cohort, this cut-off point only misclassified one paroxysmal AF individual. Although the NT-proBNP biomarker is not specific for AF and might be elevated in other cardiac diseases and renal dysfunction (33), a high sensitivity is more useful than specificity for screening purposes. NT-proBNP might be rapid and easy to use to identify all patients who need follow-up Holter monitoring since it is already widely employed as a point-of-care analysis tool for heart failure.

Although it might be suspected that elevated NT-proBNP levels were due to a higher burden of heart failure and other cardiovascular diseases in the atrial fibrillation subjects of our cohort, in the hAF group, there were few individuals with known comorbidities that could affect NT-proBNP levels. In fact, in this group, there were no cases of heart failure, and there were only differences in coronary heart disease when compared to no AF individuals. In addition, the study was carried out at the outpatient clinic, and therefore, no patient was in a situation of acute decompensated heart failure. This, combined with multivariate analysis, confirmed the independent value of NT-proBNP in diagnosing paroxysmal atrial fibrillation.

ApoC-III, vWF, ADAMTS13, uPAR, and uPA were not elevated in AF individuals in the present study. Although associated with cardioembolic stroke or AF recurrence, these biomarkers might not be useful in the context of AF screening. However, it would be interesting to perform further discovery studies to find new biomarkers that might be used in combination with NT-proBNP, increasing its specificity and sensitivity.

Interestingly, the present study showed that a higher level of NT-proBNP was correlated with higher AF burden, a conclusion based on continuous Holter monitoring. This tendency was also reflected in the distribution of NT-proBNP levels between the groups, with higher levels in individuals with a previous medical history of AF compared with individuals with hAF, as shown in Figure 1B. In fact, some studies have suggested that AF burden is related to higher stroke risk (34), and other biomarker-based models have confirmed NT-proBNP as a stroke risk surrogate (35).

Although the present study was performed in asymptomatic patients, NT-proBNP might also be useful for detecting cryptogenic stroke patients with occult AF. Recently, the negative results from clinical trials of embolic strokes of undetermined source (ESUS) patients receiving non-vitamin K antagonists (NOACs) (36, 37) indicate that this is a heterogeneous group of patients who need different management, only a minority of whom have undetected paroxysmal AF (38). In fact, the ongoing ARCADIA trial (39) will test the use of anticoagulants in ESUS patients with atrial cardiopathy using high NT-proBNP (>250 pg/ml) as one of the inclusion criteria.

Our study has some limitations. First, the participant selection was not randomized and might have suffered some selection bias: the high rates of AF might not be attributed only to the selection of high-risk patients and long-term monitoring but also to patients with more cardiac comorbidities being more prone to participate. The small sample size and the small number of patients with AF suggest that these results should be interpreted as hypothesis-generating. The protocol of the study did not include an echocardiography, missing the opportunity to establish correlations between biomarkers and left atrial diameter. Finally, the included population presented specific risk factors, and the results may not be directly applicable to populations with other combinations of risk factors. Further validation studies in larger cohorts are needed.

## Conclusions

NT-proBNP was found to be elevated in AF individuals compared to controls. This biomarker also detected cases of AF not previously known or detected by ECG. NT-proBNP may be useful as a screening biomarker in asymptomatic high-risk populations with a promising cut-off point of 95 pg/ml that requires further validation. A screening strategy based on NT-proBNP, alone or in combination with other biomarkers, might be of interest for the early initiation of anticoagulants, which could reduce cardioembolic stroke consequences.

## Data Availability Statement

The raw data supporting the conclusions of this manuscript will be made available by the authors, without undue reservation, to any qualified researcher.

## Ethics Statement

The studies involving human participants were reviewed and approved by Ethics Committee of Research Institute IDIAP Jordi Gol (P15/047/2015) and Hospital Universitari Vall d'Hebron Clinical Research Ethics Committee [PR(AG)133-2015]. The patients/participants provided their written informed consent to participate in this study.

## Author Contributions

AB, JC-E, FG-L, JB-O, and MM recruited patients. JA and AP read Holter-ECG to verify AF episodes. EP, NG, and AC performed biomarker measurements. AB, JC-E, JP, MM, and JM planned the whole project and drafted the study protocol. EP performed statistical analysis and drafted the manuscript. All authors have critically reviewed the manuscript and approved the final article version.

### Conflict of Interest

The authors declare that the research was conducted in the absence of any commercial or financial relationships that could be construed as a potential conflict of interest.
