# Supplementary material for: N-Terminal Pro B-Type Natriuretic Peptide's Usefulness for Paroxysmal Atrial Fibrillation Detection Among Populations Carrying Cardiovascular Risk Factors
Source: Front Neurol. 2019 Nov 29;10:1226. doi: 10.3389/fneur.2019.01226 (PMC6896906; doi:10.3389/fneur.2019.01226)
Supplement: Supplementary file 1 [file Table_1.DOCX]

Supplementary Material

**Supplementary Figure 1.**Boxplot distribution of ApoC-III, vWF, ADAMTS13, uPA and uPAR between AF and no AF. AF: atrial fibrillation.

**Supplementary Figure 2.**Boxplot distribution of ApoC-III, vWF, ADAMTS13, uPA and uPAR between hAF and no AF. AF: atrial fibrillation. hAF: Holter-detected group.
